# Serum n-6 Fatty Acids are Positively Associated with Growth in 6-to-10-Year Old Ugandan Children Regardless of HIV Status—A Cross-Sectional Study

**DOI:** 10.3390/nu11061268

**Published:** 2019-06-04

**Authors:** Raghav Jain, Amara E. Ezeamama, Alla Sikorskii, William Yakah, Sarah Zalwango, Philippa Musoke, Michael J. Boivin, Jenifer I. Fenton

**Affiliations:** 1 Department of Food Science and Human Nutrition, Michigan State University, East Lansing, MI 48824, USA; jainragh@msu.edu (R.J.); yakahwil@msu.edu (W.Y.); 2 Department of Psychiatry, Michigan State University, East Lansing, MI 48824, USA; ezeamama@msu.edu (A.E.E.); alla.sikorskii@hc.msu.edu (A.S.); 3 Directorate of Public Health and Environment, Kampala Capital City Authority, Kampala 00256, Uganda; skzalwango@gmail.com; 4 Makerere University-Johns Hopkins University Research Collaboration, Kampala 00256, Uganda; pmusoke@mujhu.org; 5 Departments of Psychiatry, Neurology & Ophthalmology, Michigan State University, East Lansing, MI 48824, USA; boivin@msu.edu; 6 Department of Psychiatry, University of Michigan, Ann Arbor, MI 48109, USA

**Keywords:** fatty acids, Uganda, HIV, arachidonic, omega-3, linoleic, omega-6, docosahexaenoic acid (DHA), growth, stunting

## Abstract

Fatty acids (FAs) are crucial in child growth and development. In Uganda, antiretroviral therapy (ART) has drastically reduced perinatal human immunodeficiency virus (HIV) infection of infants, however, the interplay of FAs, ART, and HIV in relation to child growth is not well understood. To investigate this, serum was collected from 240 children between 6–10 years old in Uganda and analyzed for FAs using gas-chromatography mass-spectrometry. HIV status and anthropometric measurements were taken, and relationships with FAs were assessed. No significant differences in growth parameters or serum FAs were found between HIV uninfected children with and without exposure to ART. HIV positive children had significantly lower height-for-age-*z*-scores (HAZ) than uninfected children (*p* < 0.001). HIV-positive children had higher arachidonic acid than uninfected children (*p* = 0.003). Total omega-6 FAs were significantly associated with HAZ regardless of HIV status (*p* = 0.035). Mean total omega-3 FAs (2.90%) were low in this population compared to other cohorts in Africa. These results provide reference serum FA values for 6–10-year-old children in Uganda and may be used to inform lipid supplementation programs to promote child growth. Future studies should investigate the relationships between child growth trajectories in relation to HIV status and serum FAs.

## 1. Introduction

Fatty acids are a major source of energy and are involved in a variety of bodily functions ranging from cellular signaling [1] to the development of bone mass [2]. Linoleic (LA) and α-linolenic (ALA) acids are essential omega-6 (n-6) and omega-3 (n-3) FAs, respectively, and can only be obtained through the diet. LA and ALA are elongated and further desaturated through shared enzymatic pathways to produce long chain polyunsaturated FAs (PUFAs) which are crucial to growth, development, and maintenance of cellular homeostasis [3]. Long chain PUFAs may also be obtained through the diet. The effect of FAs on child growth and development differs based on the age of the child and type of FA. Supplementation of n-3 FAs to pregnant mothers results in lower early pre-term births and higher child birthweight [4]. Additionally, n-3 FAs are required for the proper development of the central nervous system in newborn children, particularly the n-3 docosahexaenoic acid (DHA) [5]. Meanwhile, whole blood n-6 FAs are associated with positive linear growth in children between the ages of 2 and 6 years in African populations where child stunting is a public health challenge [6,7]. The majority of research on FAs and child growth focuses on children between 0–59 months of age, particularly in developing nations such as Uganda where early childhood stunting is prevalent (30.6% of children under 5 years old) [8] and has long-term, adverse health and economic implications [9,10]. However, more research is necessary to understand the relationship between FAs and growth of older children aged 6–10 years old in developing African nations.

Dietary intake plays an important role in determining the FA status of individuals. In Uganda, the diet consists mainly of foods high in carbohydrates such as cereals, sweet potatoes, and cassava. Despite access to freshwater sources, the consumption of fish, a good source of n-3 FAs, is uncommon [11]. This may lead to inadequate n-3 FA intake, causing deficits in child growth and development. According to the Food and Agriculture Organization (FAO), 89% of households in Uganda are food secure, however, nutritional awareness remains low [12]. For example, fruit and vegetable consumption by adults is low throughout Uganda, and approximately 90% of adults consume less than the recommended 5 servings of fruits and vegetables per day [13]. This may extend to children as well, negatively impacting growth. Fruits and vegetables are sources of EFAs and low consumption can result in EFA deficiency which may cause growth arrest, hinder development of the central nervous system, and compromise the immune system [14,15]. 

In Uganda, an estimated 1.3 million individuals were still living with HIV as of 2017, though the rate of new HIV infections has been decreasing since 2009 due, in part, to the implementation of antiretroviral therapies [16]. Standard antiretroviral therapy (ART) consists of several antiretroviral drugs to prevent the progression and transmission of HIV disease and is promoted by the World Health Organization in its goal to end AIDS by the year 2030 [17]. Children born with HIV are at increased risk of pre-term birth and lower overall birth size compared to uninfected children. Additionally, perinatally infected children may also have poorer fat absorption in the gastrointestinal tract than uninfected children which can affect the child FA profile [18]. Long term effects of childhood dyslipidemia include increased risk of obesity, diabetes, and hypertension [19]. Hence, ART therapies are administered to pregnant HIV positive mothers to prevent child HIV infection and its related effects. It is estimated that in 2017, ART therapies helped prevent HIV transmission to 16,000 children from their mothers, approximately double the number of children born with HIV. In Uganda, maternal HIV transmission during birth to children who are now adolescents was prevented primarily by the anti-retroviral drug nevirapine, and levels of growth stunting in children exposed but not infected with HIV due to nevirapine is similar to HIV infected infants [20]. Additionally, food insecurity is typically higher in the households of people with HIV or AIDS due increased medical costs, resulting in less resources to purchase food [21]. Therefore, there may exist a relationship between child HIV status and ART exposure, and serum FA composition, influencing growth. 

This study aimed to identify differences in FAs and growth based on HIV status in children between the ages of 6–10 years old in Kampala, Uganda. It was expected that HIV positive and ART exposed children would have an altered serum FA profile than uninfected, unexposed children which could have adversely affected their growth. These results establish reference serum FA values and may be used to develop future lipid-based interventions to support healthy growth in adolescent Ugandan children regardless of HIV status.

## 2. Materials and Methods

### 2.1. Study Context

Participants were enrolled from the Kawempe Division of Kampala, Uganda—a region with the highest prevalence of HIV (11%), compared to national prevalence of 7.3% [22]. Participants were enrolled in the context of primary care at Kawaala Health Center (KHC)—a level 3 Health Center that delivers the full range of antenatal care services, including deliveries, out-patient consulting for the general population, and the entire range of HIV/AIDS treatment and preventive services. As a birth cohort, HIV-exposed eligible children included in this study were largely born in the pre-antiretroviral therapy era. At the time of their birth, prevention of mother to child transmission of HIV was largely limited to intra-partum and neonatal single dose nevirapine [23,24] and few HIV-infected women received antiretroviral therapy for their own health.

### 2.2. Participants

Ugandan children between 6 and 10 years old and their adult primary caregivers were enrolled between March 15, 2018 and September 15, 2018 as part of a larger study of determinants of functional survival in HIV-infected/uninfected children. All measurements and sample collections were performed at enrollment. Of the 305 children in the parent study, blood samples for laboratory assessment for fatty acid measures was available in 240 that form the study base for the current analysis. Current adult caregivers were enrolled regardless of their HIV status. By design, approximately equal numbers of children with perinatally acquired HIV infection (PHIV, *n* = 102), perinatally HIV exposed but uninfected (HEU, *n* = 101) and HIV unexposed uninfected (HUU, *n* = 102) community control children were enrolled from the same hospital. Some HEU children were also exposed to ART peripartum, while PHIV children continue ART treatment postpartum.

PHIV were invited from children actively enrolled in HIV-care at KHC on a first come first serviced basis. Potentially eligible HEU children were identified through a combination of strategies that included approaching adults clinic patients identified as living with HIV through KHC antiretroviral therapy record system, identifying age-eligible HEU directly through the Early Infant Diagnosis registers, recruiting potentially eligible HEU/HUU caregiver child-pairs from the out-patient department and encouraging caregivers of confirmed HEU/HUU to recommend participation in current project to potentially eligible members of their social networks—i.e., family, friends and community. Eligibility of responding pairs were confirmed in light of study eligibility/exclusion criteria.

### 2.3. Eligibility/Exclusion Criteria

Children were eligible for the study if they had documented records of being born in a hospital/healthcare setting, were between 6 and 10 years old at enrolment, and had available health records from which data regarding their general health, HIV status of index child at birth and the HIV status of biological mother were objectively confirmed. Children were excluded if born in non-clinic settings, or if they were children of caregivers without official birth records and/or missing antenatal register/delivery medical records as the HIV-status of the birth mother and HIV-status of the index child at birth could not be reliably ascertained.

### 2.4. Statement of Ethical Approval

The study protocol was approved by the research ethics review committees of Michigan State University (IRB Protocol#: 16-828), Makerere University College of Health Sciences, School of Medicine (Protocol REC REF# 2017-017) and the Uganda National Council for Science and Technology (Protocol #: SS 4378). All caregivers gave written informed consent and children provided assent for study participation.

### 2.5. Outcome Variables: Height-for-Age, Weight-for-Age and Body Mass Index for Age

Anthropometric measurements were performed by trained health care professionals. Height and weight measurements were taken in triplicate and recorded as the mean of three measures. Height was measured with children directly looking forward and in contact with a flat wall. Specifically, children were not wearing shoes and the back of their feet, calves, bottom, upper back and back of head was in contact with the wall. Each child’s height is determined in centimeters by measuring the distance from the ground to a mark against the wall is made directly from the top of children’s head. Weight was measured in kilograms on a calibrated beam balance scale (Seca Classic Beam Scale, Model#700, Seca Inc., China, CA, USA). For analytic purposes, growth parameters are analyzed as continuous outcome variables with height-for-age *z*-score (HAZ), weight-for-age *z*-score (WAZ), and BMI-for-age *z*-score (BAZ) calculated using WHO AnthroPlus (Geneva, Switzerland) [25].

### 2.6. Hemoglobin, CD4 Cell Count, and HIV Assessment

Hemoglobin in grams per deciliter was assessed as part of complete blood count. Absolute T-cell lymphocyte count in cells/micro liter was measured using a fluorescence-activated cell sorting (FACS) Calibur flow cytometer (Becton-Dickinson, San Jose, CA, USA). Child perinatal HIV status was defined on the basis of HIV mother-to-child transmission as PHIV, HEU and HUU. PHIV and HEU status were objectively determined prior to the 18th month of life on the basis of a positive and negative DNA-polymerase chain reaction, respectively. For both HEU and HUU, HIV-status at enrolment was confirmed on the basis of a negative HIV-rapid diagnostic test [26,27].

### 2.7. Primary Exposure: Serum Fatty Acid

At the time of enrollment, venous blood was collected. Serum fractions were separated from venous blood after collection and stored at −80 °C. Serum samples was shipped on dry ice to the US for fatty acid analysis. Fatty acids were extracted and methylated from serum as described by Masood et al. (2005) [28]. Briefly, 200 μL of serum and 2 mL of a 1.8:0.2 (*v*/*v*) solution of freshly prepared methanol (0.01% (*w*/*v*) butylated-hydroxy-toluene):acetyl chloride containing C18d35 internal standard (Sigma-Aldrich, St. Louis, MO, USA) were mixed in a test tube with 400 uL hexane and heated at 100 °C for 1 h. The mixture was cooled to room temperature and neutralized with 2 mL of 5% (*w*/*v*) sodium bicarbonate. Fatty acid methyl esters (FAMEs) were extracted to a new tube using 2 × 2 mL portions of hexane and dried under nitrogen. FAMEs were resuspended in isooctane and stored in capped GC vials at −20 °C until GC/MS analysis. All reagents used were HPLC-grade or higher and purchased from Sigma-Aldrich unless otherwise specified. Fatty acid values are presented as percent of total FAs.

For analytic purposes, fatty acid measures were operationally defined as: (a) a continuous covariate and (b) as a categorical covariate. First, as a continuous variable, each FA was normalized by the sample wide standard deviation in order to estimate differences in growth per standard deviation increase in a given FA. Of note, continuous variables assume a linear functional form in relationship to growth measures which may not hold in throughout the range of fatty acid values. Hence, to relax linear functional form assumption for continuous measures, FA was also analyzed FA as categorical covariate. Four categories of approximately equal size were defined based on the quartiles of respective fatty acids in our sample and differences in growth outcomes were calculated for three lower quartiles compared to the highest quartile.

### 2.8. GC/MS Analysis

A Clarus 600/680 gas chromatograph/mass spectrometer (Perkin-Elmer, Waltham, MA, USA) (GC/MS) was used for FAME analysis. The GC was equipped with a DB-23 (30 m length, 25 mm ID) column (Agilent, Santa Clara, CA, USA) and used helium as carrier gas. The GC temperature profile was as follows: 100 °C, ramp 8 °C/min to 200 °C, ramp 2.5 °C/min to 220 °C, ramp 10 °C/min to 240 °C, hold for 1 min. Selective ion monitoring was used to improve FA resolution. The Supelco 37 component FAME mix (Sigma-Aldrich) was used to create standard curves for all FAMEs except palmitelaidic, mead, n-6 docosatetraenoic (DTA), n-6 docosapentaenoic (DPA n-6), and n-3 docosapentaenoic (DPA n-3) acid methyl esters, which are not present in the Supelco mix and had to be purchased separately from Cayman Chemical (Ann Arbor, MI, USA). TargetLynx version 4.0.1 (Waters Corporation, Milford, MA, USA) was used for all GC/MS data analysis. 

### 2.9. Statistical Analysis

As part of descriptive analyses, mean and standard deviations of continuous variables and number and proportion for categorical variables are presented in the overall sample and by HIV status to describe growth measures, fatty acid levels and socio-demographic characteristics. The mean and standard error of the mean (SEM) were reported for all FAs and ANOVA with Tukey’s post-hoc test was used to assess differences based on HIV status for all continuous variables. The *Χ*^2^ test was used for comparison of all categorical variables. Multivariable linear regression for each growth measure was implemented in relationship to FAs with adjustment for child HIV status, child hemoglobin, the caregiver’s sex, the caregiver’s education, and the caregiver’s quality scale as covariates. Mean differences and associations were considered statistically significant if *p* < 0.05. Statistical analyses of data were performed using R version 3.4.3 (R Foundation for Statistical Computing, Vienna, Austria).

To evaluate the potential for heterogeneity in relationship of FA to growth measures by HIV status, an interaction term between HIV status and FA (HIV*FA) quartile was introduced in multivariable models including respective individual measures. Wherever *p*-value for the interaction term was <=0.10, the effect of FAs on growth was explored within each HIV stratum. All regression analyses of FAs as categorical covariates was implemented in Statistical Analysis Software (SAS) version 9.4 (SAS Institute Inc., Cary, NC, USA).

## 3. Results

### 3.1. Population Characteristics

Information on the mean ± sd of population demographics with a breakdown based on HIV status is presented in Table 1. 

The average age of the children was 8.06 ± 1.53 years and did not differ among children in different HIV groups (*p* = 0.524). A similar number of male (52.5%) and female (47.5%) participants were enrolled. The mean height, weight, and hemoglobin level was 124 cm, 23.4 kg and 13.1 g/dL, respectively. The CD4 count of PHIV children was 1340 cells per microliter. 

### 3.2. Population Serum Fatty Acid Levels and Breakdown by HIV Status

The mean and SEM of individual fatty acid proportions of the children is presented in Table 2. 

Total saturated FAs (SFAs) accounted for 41.9 ± 0.300% of the total FAs and did not differ due to HIV status (*p* = 0.387). Oleic acid, the most abundant monounsaturated FA (MUFA), made up 3.39 ± 0.050% of total FAs and also did not differ due to HIV status (*p* = 0.148). Though total n-6 FAs (49.5 ± 0.328%) did not differ between HIV groups (*p* = 0.446), the essential FA linoleic acid was significantly higher in HEU (37.7 ± 0.584%) than the PHIV (35.4 ± 0.591%) group (Tukey’s *p* = 0.016). In contrast, the n-6 FA arachidonic acid (AA) was significantly higher in PHIV children (12.5 ± 0.262%) than HEU (11.3 ± 0.257%, Tukey’s *p* = 0.003) and HUU children (11.8 ± 0.232%, Tukey’s *p* = 0.026). n-3 FAs did not differ significantly between HIV groups. HIV related differences in growth outcomes of the children are presented in Table 3. There were no differences in BAZ and WAZ between the HIV groups. However, HAZ for PHIV children was lower in comparison with HUU (difference = −0.48, *p* = 0.002) and HEU (difference = −0.49, *p* = 0.005) groups. There were no differences in any growth measure for HEU compared to HUU (all *p* ≥ 0.472, Table 3).

### 3.3. Relationship between Fatty Acids and Growth

Mean differences in growth parameters- HAZ, WAZ, and BAZ, per standard deviation increase in respective FA measures are presented in Table 4. Total SFAs were inversely related to HAZ (*p* = 0.027), with a 0.16 *z*-score decrease in HAZ per standard deviation increase in total SFAs. In contrast, total n-6 FAs were positively associated with HAZ (*p* = 0.035). The R^2^ adjusted value for HAZ regressions were between 4–7%. Total MUFAs were inversely associated with WAZ (*p* = 0.033), and palmitelaidic acid was inversely related to BAZ (*p* = 0.021). The R^2^ adjusted values for WAZ and BAZ regressions were between −4% and 3%.

Mean differences in growth measures for the three lower FA quartiles relative to the highest FA quartile are presented in Table 5. Children in the lowest quartile for both DHA (β = −0.39; *p* = 0.029) and LA (β = −0.38; *p* = 0.028) had a lower WAZ relative to their peers in the highest quartile of the respective FAs. Likewise, being in the lowest quartile for DHA (β = −0.42; *p* = 0.038), total n-3 (β = −0.45; *p* = 0.029), and LA (β = −0.43; *p* = 0.005) relative to being in the highest quartile for the respective FAs was associated with significantly lower HAZ. On the other hand, children in the lower two quartiles of the n-6 FA eicosadienoic acid (β = 0.35 to 0.38; *p* ≤ 0.047) had a higher BAZ score than peers in the highest quartile. Additionally, being in quartiles 2–3 vs. the highest quartile for oleic acid was associated with higher HAZ (β = 0.045, 0.038; *p* < 0.05 for quartiles 2–3, respectively).

The interaction term between HIV status and oleic acid in quartile regressions (Table 5) was lower than *p* = 0.100 for both WAZ and BAZ regressions and almost significant for the HAZ regression (*p* = 0.101), hence differences in growth between HIV groups was explored in relation to oleic acid quartiles in Table 6. Interestingly, significant (*p* < 0.05) relationships between oleic acid quartiles and all three growth parameters was present for HIV positive and negative children, but not for exposed children. Additionally, all significant relationships were positive, indicating that HIV positive and negative children not in the highest quartile for oleic acid had better growth outcomes than children in the highest oleic acid quartile.

## 4. Discussion

In this study, the serum FA profiles of 240 children from the Kawempe Division of Kampala, Uganda were characterized and related to anthropometric growth measurements. Total n-6 FAs was positively associated with linear growth, measured by HAZ. This is in agreement with a previous report of children six years old and under from Tanzania [6]. Results from several studies in HEU children indicate exposure to HIV and/or early exposure to ART causes slower growth during infancy when compared to HUU children [29,30,31]. Our data suggests no significant differences in growth measures were detected between HEU and HUU for 6–10-year-old Ugandan children in this study. Additionally, WAZ and BAZ did not differ among HEU, HUU, and PHIV children, though HAZ was significantly lower in PHIV children than HEU and HUU children. This may be due to lower levels of the most abundant n-6 FA, LA, in PHIV children than the other groups. It has previously been reported that there is increased turnover of LA into AA in PHIV compared to HUU children [32]. Indeed, the AA content of PHIV children was higher than HUU (and HEU) children. Arachidonic acid is the primary substrate for oxygenation enzymes which generate eicosanoids, modulators of inflammation and resolution in the body. Certain AA-derived prostaglandins, a class of eicosanoids, have been shown to prevent HIV infection of immune cells [33]. Therefore, in the PHIV group, increased need for AA-derived metabolites may be leading to higher LA to AA turnover, lowering LA availability for other functions, such as bodily growth. There are a few studies that have looked at the long-term differences in growth trajectories of HEU and HUU children. We found no differences in growth between HEU and HUU groups. However, the metabolic implications of early ART exposure for the long-term growth of HEU children is unknown [34] and deserving of specific future investigation.

Interestingly, EFA deficiency was not detected in this population. EFA deficiency is determined by the triene-to-tetraene ratio, or mead acid/AA, and a ratio of 0.02 is a clinical marker of EFA deficiency [35]. The lack of EFA deficiency in this population indicates that these children are receiving adequate amounts of EFAs through their diet [13]. This may be a result of Kampala being an urban area and therefore a larger variety of foods are available for consumption. Notably, the triene-to-tetraene ratio was inversely associated with HAZ, WAZ, and BAZ, emphasizing the importance of EFAs in the diet for growth outcomes. No other FA or FA index was significantly associated with all three growth outcomes. Surprisingly, total MUFAs were inversely related to WAZ (β = 0.15, *p* = 0.033). This was largely driven by oleic acid, the most abundant MUFA. The middle two quartiles of oleic acid were associated with higher HAZ and BAZ, and all oleic quartiles were associated with higher WAZ when compared to the highest quartile of oleic acid. This was unexpected because oleic acid is recognized as being important in brain development, especially during myelination [36], and increases membrane fluidity in cells. Furthermore, the mean oleic acid and total MUFA serum composition of the children in Uganda (3.39 and 5.65%, respectively) is low when compared to children from Ghana or Tanzania (approximately 20% oleic and 22% total MUFAs) [6,7]. It is possible that production of oleic acid in Ugandan children results from increased elongation of the saturated FA palmitic acid to stearic acid followed by desaturation to oleic acid due to high SFA availability. Hence, increases in oleic acid content may be the result of the body trying to process high SFA rather than due to low dietary intake. This is supported by higher total SFA in the current Ugandan population (41.9%) than the Ghanaian or Tanzanian populations (37.5 and 40.42% respectively). All three studies analyzed the same FAs hence the values are comparable. These observations may have broader implications for child growth and development supported by the inverse relationship observed between total SFAs and HAZ/WAZ. Given the nature of the cross-sectional design, no cause-effect relationship can be established and further investigation into this relationship is warranted. 

Total n-3 FAs were low in the Ugandan population when compared to that of 7–9-year-olds in Zimbabwe (2.90 vs. 3.13, respectively). This may be due to low consumption of foods rich in n-3 FAs as the diet is an important determinant of n-3 status, however no conclusion can be made due to lack of information on the children’s diet. The highly unsaturated FA (HUFA) ratio was higher in the Zimbabwean than Ugandan population (18.4 vs. 17.0, respectively) [37]. The HUFA ratio here represents the percent of long chain (20+ carbon) PUFAs that are n-3 FAs, and it is gaining interest as a marker to estimate eicosanoid levels intensity in the body [38]. In the current context, a lower HUFA ratio is indicative of increased n-6 derived eicosanoids which are generally considered pro-inflammatory. It should be noted that the Zimbabwe group researchers excluded nervonic and lignoceric acid from their MUFA and SFA analyses, and mead acid from their total long chain PUFA calculation. Lignoceric and nervonic acids were in low abundance (<1%) in our study and minimally affected other FA values. Levels of mead acid in the Ugandan population were negligible (<0.10%) in comparison to the other long chain PUFAs, notably DHA (2.11%) and AA (11.8%), and did not affect the HUFA ratio calculation. The n-3 FAs in this cohort were also lower than that of young children from Northern and Southern Ghana (3.47 and 7.15% of total FAs, respectively), and both Ghanaian studies analyzed the same FAs as the current study [7,39]. Specifically, the omega-3 FAs are important for proper development and functioning of the central nervous system, and metabolites of the long chain n-3 FAs, EPA and DHA, are involved in maintaining cellular homeostasis, particularly in the resolution of inflammation [40]. In fact, supplementation of DHA in the diet of 7–9-year-old school children in South Africa improved cognitive test performance [41]. Additionally, though differences in n-3 FAs among the HIV groups was not detected, it is possible that n-3 supplementation to HIV positive persons may alleviate virus-related inflammation, providing further health benefits to PHIV children [42]. Given the importance of n-3 FA in growth, cognition and inflammation and the relatively lower baseline n-3 levels in this population, all groups may benefit from n-3 supplementation.

In this study, no differences in total SFAs, MUFAs or PUFAs were observed between PHIV and HUU children, the SFA stearic acid was higher in PHIV children and, as previously discussed, AA was higher and LA lower in PHIV vs. HUU children. This agrees with previous reports. In fact, the overall FA profile of HIV positive individuals is altered compared to uninfected individuals, resulting in higher SFAs and lower MUFAs and PUFAs [43]. Treatment with ART is associated with increased high-density lipoprotein (HDL cholesterol) [44], and the increase in stearic acid may be attributed to increased incorporation of the neutral SFA into lipoproteins. It is likely that more dramatic differences in FA classes were not observed in this population due to adequate management of the disease as ART therapy helps normalize the lipid profile of HIV positive individuals to similar, though not the same, levels as uninfected people [43]. No significant differences in the serum FA composition of HEU and HUU groups was detected. In HEU infants, lower insulin secretion has been observed when compared to HUU, likely altering their serum FA composition [45,46]. These results indicate that changes in serum FA composition during infancy may not persist in HEU children as they grow older.

The authors acknowledge there are limitations to this study. Information on the diet of the children is unavailable, hence dietary effects on child FA composition could not be discussed. This population is from an urban area and the results may not be representative of other adolescent populations in rural Uganda due to dietary and lifestyle differences. Participants were recruited in the context of primary care, however acutely ill children were given appointments to return for study-related evaluation when the child was well. Because HIV-infected children are systematically linked to the clinic for HIV-care and were less likely to be acutely ill, this element of design may have resulted in muted contrasts for HIV-uninfected relative to HIV-infected children on nutritional measures. In addition, the cross-sectional design of this study limits our ability to infer that relationships between fatty acid deficiency and growth outcomes are causal. There are many strengths including the large sample size, which allowed for meaningful evaluation of anthropometric growth parameters in relation to several factors. In this study, we systematically characterized the serum FA related differences in growth Ugandan children with and without perinatal HIV exposure/infection during early school-age years (i.e., age 6–10 years), while also controlling for sociodemographic confounders such as child caregiver education. Few studies have focused on growth of children between the ages of 6–10 years in sub-Saharan Africa. The data reported here may serve as reference values for future researchers. Furthermore, this study provides valuable information for comparison of not only HIV infected and uninfected individuals, but also of perinatally exposed, uninfected children. The information presented here may be used to tailor nutrient supplementation programs to the population in order to improve child growth, development, and health outcomes.

## 5. Conclusions

The growth and serum FA profile of 6-to-10-year old Ugandan children that were PHIV, HEU, or HUU was assessed. No differences in growth or FA profile was observed between HEU and HUU children, therefore perinatal exposure to ART may not affect long-term growth and development. However, HAZ was lower in PHIV vs. HEU and HUU children, indicating perinatal HIV infection may cause a reduction in growth over time. PHIV children had lower LA and higher AA and stearic acid compared to HEU and HUU. Total n-3s were low across the entire population when compared to other African cohorts. Total n-6 FAs were positively associated with HAZ. This was consistent with previous reports. Surprisingly, higher MUFAs was inversely associated with HAZ, WAZ, and BAZ. This was an unexpected finding and warrants further investigation. These results may be used as reference values for future studies on lipid supplementation to support growth in the population. Additionally, future studies should focus on FA differences and child growth trajectories in relation to perinatal HIV status as literature on the topic is limited.

## Figures and Tables

**Table 1:** Socio-demographic and clinical description (mean ± sd) of early school-aged children with and without perinatal HIV infection/exposure from Kampala, Uganda ^1^.

Demographic	Regardless of HIV Status	HIV Unexposed Uninfected	HIV Exposed Uninfected	Perinatally HIV Infected	*p*-Value ^2^
	(*n* = 240)	(*n* = 79)	(*n* = 77)	(*n* = 84)	
Age (years)	8.06 ± 1.53	8.14 ± 1.51	7.89 ± 1.47	8.13 ± 1.61	0.509
Sex (*n* = 240)					
Male	126 (53%)	45 (57%)	36 (47%)	45 (54%)	0.430
Female	114 (47%)	34 (43%)	41 (53%)	39 (46%)	
Height (cm)	124 ± 9.97	125 ± 9.68	124 ± 10.5	122 ± 9.67	0.175
Weight (kg)	23.4 ± 5.11	24.0 ± 5.38	23.2 ± 4.81	23.0 ± 5.14	0.509
Hb (g/dL)	13.1 ± 1.30	13.2 ± 1.05	13.1 ± 1.52	12.9 ± 1.29	0.191
BAZ	−0.55 ± 1.05	−0.59 ± 1.14	−0.60 ± 1.04	−0.48 ± 0.97	0.740
HAZ	−0.22 ± 1.11	−0.02 ± 1.03	0.00 ± 1.24	−0.61 ± 0.95	**0.003**
WAZ	−0.50 ± 1.02	−0.38 ± 0.97	−0.40 ± 1.09	−0.70 ± 0.99	0.081
CD4 count (cells/uL)	-	-	-	1340 ± 898	-
Caregiver Education (*n* = 233) ^3^					
none-some primary	94 (39%)	24 (30%)	38 (49%)	32 (38%)	**0.023**
completed primary	48 (20%)	16 (20%)	17 (22%)	15 (18%)	
some or completed O levels	72 (30%)	25 (32%)	19 (25%)	28 (33%)	
≥some A levels	19 (8%)	12 (15%)	1 (1%)	6 (7%)	

^1^ Hb, hemoglobin; BAZ, BMI-for-age *z*-score; HAZ, height-for-age *z*-score; WAZ, weight-for-age *z*-score. ^2^ ANOVA used to compare variable means amongst HIV groups except for the categorical variables ‘Sex’ and ‘Education’ for which *Χ*^2^ test was used. ^3^ Grading system based on old U.K. standards. O = ordinary level examinations taken until 16 years of age, A = advanced level examinations for students continuing education until 18 years. Not disclosed by seven caregivers. Bolding indicates statistical significance (*p* < 0.05).

**Table 2:** Basic description of serum fatty acid proportions (mean ± SEM) in 6–10-year-old Ugandan children with and without perinatal HIV infection/exposure. ^1^

Class	Fatty acid	Regardless of HIV Status	HIV Unexposed Uninfected	HIV Exposed Uninfected	Perinatally HIV Infected	*p*-Value ^5^
SFA	Myristic	0.665 ± 0.022	0.642 ± 0.028	0.679 ± 0.044	0.673 ± 0.040	0.761
	Palmitic	29.1 ± 0.213	29.4 ± 0.319	29.4 ± 0.452	28.6 ± 0.325	0.229
	Stearic	11.0 ± 0.137	10.7 ± 0.230 ^b^	10.4 ± 0.229 ^b^	11.8 ± 0.226 ^a^	**<0.001**
	Arachidic	0.198 ± 0.004	0.192 ± 0.008 ^b^	0.184 ± 0.008 ^b^	0.215 ± 0.007 ^a^	**0.009**
	Behenic	0.579 ± 0.014	0.571 ± 0.021	0.561 ± 0.028	0.602 ± 0.026	0.484
	Lignoceric	0.318 ± 0.009	0.336 ± 0.015	0.303 ± 0.017	0.316 ± 0.013	0.303
	Total SFA	41.9 ± 0.300	41.8 ± 0.483	41.6 ± 0.586	42.3 ± 0.494	0.645
MUFA	Palmitoleic	1.03 ± 0.032	1.05 ± 0.049	1.06 ± 0.058	0.979 ± 0.058	0.547
	Palmitelaidic	0.072 ± 0.003	0.063 ± 0.004	0.071 ± 0.005	0.080 ± 0.005	**0.039**
	Oleic	3.39 ± 0.050	3.27 ± 0.075	3.40 ± 0.101	3.50 ± 0.084	0.178
	Eicosenoic	0.364 ± 0.019	0.338 ± 0.011	0.406 ± 0.057	0.349 ± 0.010	0.311
	Nervonic	0.798 ± 0.016	0.856 ± 0.029 ^a^	0.795 ± 0.028 ^a^	0.746 ± 0.026 ^b^	**0.019**
	Total MUFA	5.65 ± 0.075	5.58 ± 0.114	5.73 ± 0.142	5.65 ± 0.133	0.732
n-6 FA	Linoleic	36.8 ± 0.347	37.3 ± 0.601 ^ab^	37.7 ± 0.584 ^a^	35.4 ± 0.591 ^b^	**0.013**
	Linoelaidic	0.008 ± 0.001	0.007 ± 0.001 ^b^	0.006 ± 0.001 ^b^	0.010 ± 0.001 ^a^	**0.021**
	GLA	0.061 ± 0.003	0.056 ± 0.005	0.053 ± 0.004	0.072 ± 0.006	**0.021**
	Eicosadienoic	0.205 ± 0.005	0.193 ± 0.008 ^b^	0.190 ± 0.008 ^b^	0.230 ± 0.010 ^a^	**0.001**
	DGLA	0.563 ± 0.017	0.491 ± 0.021 ^b^	0.452 ± 0.022 ^b^	0.734 ± 0.031 ^a^	**<0.001**
	Arachidonic	11.8 ± 0.148	11.6 ± 0.232 ^b^	11.3 ± 0.257 ^b^	12.5 ± 0.262 ^a^	**0.003**
	DTA	0.018 ± 0.001	0.017 ± 0.001 ^b^	0.016 ± 0.001 ^b^	0.021 ± 0.001 ^a^	**<0.001**
	DPA n-6	0.014 ± 0.001	0.013 ± 0.001 ^b^	0.011 ± 0.001 ^b^	0.019 ± 0.001 ^a^	**<0.001**
	Total n-6	49.5 ± 0.328	49.6 ± 0.554	49.8 ± 0.610	49.0 ± 0.548	0.574
n-3 FA	ALA	0.262 ± 0.010	0.269 ± 0.017	0.265 ± 0.020	0.252 ± 0.016	0.765
	EPA	0.459 ± 0.021	0.460 ± 0.031	0.452 ± 0.031	0.465 ± 0.045	0.968
	DPA n-3	0.067 ± 0.003	0.066 ± 0.005	0.062 ± 0.005	0.072 ± 0.004	0.311
	DHA	2.11 ± 0.058	2.12 ± 0.111	2.03 ± 0.092	2.18 ± 0.100	0.543
	O3I	3.28 ± 0.094	3.29 ± 0.169	3.16 ± 0.142	3.38 ± 0.173	0.631
	Total n-3	2.90 ± 0.077	2.91 ± 0.138	2.81 ± 0.122	2.97 ± 0.141	0.672
	Mead acid	0.085 ± 0.004	0.072 ± 0.004 ^b^	0.074 ± 0.004 ^b^	0.108 ± 0.008 ^a^	**<0.001**
	Total PUFA ^2^	52.4 ± 0.328	52.6 ± 0.513	52.7 ± 0.646	52.1 ± 0.545	0.714
	Total HUFA ^3^	15.1 ± 0.203	14.8 ± 0.317 ^b^	14.4 ± 0.339 ^b^	16.1 ± 0.366 ^a^	**0.002**
	HUFA ratio ^4^	17.0 ± 0.329	17.3 ± 0.649	17.3 ± 0.567	16.6 ± 0.492	0.576
	T/T ratio	0.007 ± 0.000	0.006 ± 0.000 ^b^	0.007 ± 0.000 ^b^	0.009 ± 0.001 ^a^	**0.002**
	n-6/n-3 ratio	20.6 ± 0.770	20.6 ± 1.15	22.1 ± 1.76	19.2 ± 1.05	0.331

^1^ All values expressed as % of total fatty acids. FA, fatty acid; SFA, saturated FA; MUFA, monounsaturated FA; n-6, omega-6; GLA, γ-linolenic acid; DGLA, dihomo-γ-linolenic acid; DTA, docosatetraenoic acid; DPA, docosapentaenoic acid; n-3, omega-3; ALA, α-linolenic acid; EPA, eicosapentaenoic acid; DHA, docosahexaenoic acid; O3I, omega-3 index; PUFA, polyunsaturated FA; HUFA, highly unsaturated FA; T/T, triene-to-tetraene. ^2^ Total PUFA is sum of total n-6, total n-3, and mead acid. ^3^ Total HUFA is sum of DGLA, Arachidonic, DTA, DPA n-6, EPA, DPA n-3, DHA, and Mead acid. ^4^ HUFA ratio is the sum of n-3 HUFAs divided by Total HUFA×100. ^5^ *P*-value from ANOVA comparing mean FA values across all HIV groups. Letters denote results of Tukey’s post-hoc test such that groups without a common letter are significantly different (*p* < 0.05). Bolding indicates statistical significance (*p* < 0.05).

**Table 3:** HIV-related differences in growth parameters of 6–10-year-old Ugandan children. ^1^

Growth	Perinatally HIV-Infected vs. Unexposed Uninfected	Perinatally HIV-Infected vs. HIV Exposed Uninfected	HIV Exposed Uninfected vs. Unexposed Uninfected
Outcome	Difference (*p*-value)	Difference (*p*-value)	Difference (*p*-value)
HAZ	−0.48 (**0.002**)	−0.49 (**0.005**)	0.01 (0.936)
WAZ	−0.17 (0.283)	−0.21 (0.196)	0.04 (0.816)
BAZ	0.23 (0.194)	0.10 (0.581)	0.14 (0.472)

^1^ HAZ, height-for-age *z*-score; WAZ, weight-for-age *z*-score; BAZ, BMI-for-age *z*-score. Bolding indicates statistical significance (*p* < 0.05).

**Table 4:** Mean difference in height-for-age, weight-for-age and bod mass index for age *z*-scores per standard deviation increase in respective fatty acid proportions among 6–10 years old Ugandan children with and without perinatal HIV infection ^1^.

Class	Fatty Acid	Height-for-Age *z*-Score		Weight-for-Age *z*-Score		Body Mass Index-for-Age *z*-Score	
		B ± SE	*p*-Value	B ± SE	*p*-Value	B ± SE	*p*-Value
**SFA**	Myristic	−0.07 ± 0.07	0.334	−0.10 ± 0.07	0.156	−0.09 ± 0.07	0.204
	Palmitic	−0.14 ± 0.07	0.053	−0.09 ± 0.07	0.206	0.00 ± 0.07	0.956
	Stearic	−0.12 ± 0.08	0.135	−0.07 ± 0.07	0.365	0.01 ± 0.08	0.898
	Arachidic	−0.09 ± 0.07	0.226	−0.03 ± 0.07	0.702	0.05 ± 0.07	0.512
	Behenic	−0.08 ± 0.07	0.254	−0.00 ± 0.07	0.987	0.09 ± 0.07	0.218
	Lignoceric	−0.07 ± 0.07	0.314	−0.01 ± 0.07	0.862	0.05 ± 0.07	0.496
	Total SFA	−0.16 ± 0.07	**0.027**	−0.10 ± 0.07	0.158	0.01 ± 0.07	0.927
MUFA	Palmitoleic	−0.05 ± 0.07	0.454	−0.07 ± 0.07	0.314	−0.06 ± 0.07	0.422
	Palmitelaidic	−0.05 ± 0.07	0.507	−0.13 ± 0.07	0.052	−0.17 ± 0.07	**0.021**
	Oleic	−0.08 ± 0.07	0.248	−0.13 ± 0.07	0.060	−0.12 ± 0.07	0.079
	Eicosenoic	−0.06 ± 0.07	0.399	−0.04 ± 0.07	0.613	0.00 ± 0.07	0.970
	Nervonic	−0.16 ± 0.07	**0.028**	−0.08 ± 0.07	0.258	0.04 ± 0.07	0.546
	Total MUFA	−0.13 ± 0.07	0.073	−0.15 ± 0.09	**0.033**	−0.11 ± 0.07	0.142
n-6 FA	Linoleic	0.13 ± 0.08	0.100	0.09 ± 0.07	0.233	0.01 ± 0.08	0.919
	Linoelaidic	0.06 ± 0.07	0.386	0.04 ± 0.07	0.591	−0.00 ± 0.07	0.982
	GLA	−0.03 ± 0.07	0.726	−0.08 ± 0.07	0.248	−0.10 ± 0.07	0.186
	Eicosadienoic	−0.08 ± 0.08	0.285	−0.13 ± 0.07	0.073	−0.12 ± 0.08	0.126
	DGLA	−0.01 ± 0.08	0.892	−0.05 ± 0.08	0.513	−0.07 ± 0.08	0.395
	Arachidonic	0.08 ± 0.08	0.308	0.08 ± 0.07	0.279	0.05 ± 0.08	0.508
	DTA	−0.02 ± 0.08	0.806	−0.04 ± 0.07	0.613	−0.05 ± 0.08	0.548
	DPA n-6	0.04 ± 0.08	0.641	0.04 ± 0.07	0.578	0.02 ± 0.08	0.765
	Total n-6	0.16 ± 0.07	**0.035**	0.11 ± 0.07	0.103	0.02 ± 0.07	0.749
n-3 FA	ALA	0.05 ± 0.07	0.478	−0.03 ± 0.07	0.668	−0.09 ± 0.07	0.186
	EPA	0.03 ± 0.07	0.677	−0.03 ± 0.07	0.720	−0.06 ± 0.07	0.390
	DPA n-3	0.09 ± 0.08	0.212	0.01 ± 0.07	0.897	−0.08 ± 0.08	0.293
	DHA	0.11 ± 0.07	0.118	0.09 ± 0.07	0.216	0.03 ± 0.07	0.717
	O3I	0.10 ± 0.07	0.169	0.06 ± 0.07	0.376	0.00 ± 0.07	0.968
	Total n-3	0.10 ± 0.07	0.152	0.05 ± 0.07	0.433	−0.01 ± 0.07	0.865
	Mead	−0.14 ± 0.09	0.126	−0.20 ± 0.08	**0.015**	−0.18 ± 0.09	0.406
	Total PUFA	0.18 ± 0.07	**0.015**	0.12 ± 0.07	0.074	0.02 ± 0.07	0.795
	Total HUFA	0.09 ± 0.08	0.215	0.07 ± 0.07	0.301	0.03 ± 0.08	0.699
	HUFA ratio	0.11 ± 0.07	0.147	0.07 ± 0.07	0.348	0.00 ± 0.07	0.982
	T/T ratio	−0.17 ± 0.08	**0.039**	−0.22 ± 0.08	**0.006**	−0.17 ± 0.08	**0.048**
	n-6/n-3 ratio	−0.04 ± 0.07	0.572	−0.03 ± 0.07	0.689	−0.00 ± 0.07	0.961

^1^ All individual fatty acid values were divided by the standard deviation of the fatty acid prior to being regressed. Beta values are interpreted as “change in growth parameter per standard deviation change in fatty acid.” FA, fatty acid; SFA, saturated FA; MUFA, monounsaturated FA; n-6, omega-6; GLA, γ-linolenic acid; DGLA, dihomo-γ-linolenic acid; DTA, docosatetraenoic acid; DPA, docosapentaenoic acid; n-3, omega-3; ALA, α-linolenic acid; EPA, eicosapentaenoic acid; DHA, docosahexaenoic acid; O3I, omega-3 index; PUFA, polyunsaturated FA; HUFA, highly unsaturated FA; T/T, triene-to-tetraene. Bolding indicates statistical significance (*p* < 0.05).

**Table 5:** Mean difference in growth measures for lower vs. highest quartiles of respective fatty acid proportions among 6–10 years old Ugandan children with and without Perinatal HIV infection ^1^.

Fatty Acid	HAZ				WAZ				BAZ			
	Q1	Q2	Q3	HIV*FA^2^	Q1	Q2	Q3	HIV*FA	Q1	Q2	Q3	HIV*FA
Palmitic	0.29 (0.166)	0.00 (0.973)	−0.03 (0.862)	0.283	0.27 (0.183)	0.12 (0.526)	0.15 (0.416)	0.365	0.14 (0.495)	0.12 (0.532)	0.26 (0.162)	0.475
Total SFA	0.30 (0.126)	0.18 (0.385)	−0.18 (0.332)	0.984	0.27 (0.137)	0.27 (0.165)	−0.15 (0.354)	0.813	0.14 (0.490)	0.26 (0.178)	−0.02 (0.893)	0.481
Oleic	0.35 (0.055)	**0.45 (0.043)**	**0.38 (0.020)**	0.101	**0.34 (0.049)**	**0.51 (0.011)**	**0.46 (0.008)**	0.051	0.20 (0.222)	**0.50 (0.002)**	**0.37 (0.035)**	0.088
Eicosenoic	0.34 (0.108)	0.01 (0.981)	−0.21 (0.233)	0.905	0.31 (0.121)	0.12 (0.528)	0.00 (0.986)	0.523	0.18 (0.393)	0.19 (0.326)	0.22 (0.242)	0.318
Mead acid	0.33 (0.074)	0.06 (0.763)	−0.09 (0.632)	0.183	0.33 (0.086)	0.22 (0.197)	0.07 (0.725)	0.628	0.22 (0.270)	0.29 (0.087)	0.20 (0.331)	0.412
Nervonic	0.23 (0.282)	0.08 (0.707)	−0.03 (0.865)	0.275	0.14 (0.475)	0.03 (0.897)	0.06 (0.747)	0.547	−0.05 (0.779)	−0.08 (0.699)	0.10 (0.621)	0.548
Total MUFA	0.31 (0.075)	**0.52 (0.005)**	0.17 (0.322)	0.850	0.26 (0.146)	**0.61 (0.001)**	0.28 (0.107)	0.500	0.09 (0.678)	**0.46 (0.020)**	0.29 (0.134)	0.592
EPA	−0.19 (0.342)	**−0.42 (0.028)**	−0.38 (0.077)	0.164	0.10 (0.618)	−0.14 (0.469)	−0.29 (0.170)	0.562	0.31 (0.103)	0.22 (0.253)	−0.10 (0.253)	0.766
DHA	**−0.42 (0.038)**	−0.08 (0.691)	0.12 (0.559)	0.811	**−0.39 (0.029)**	−0.06 (0.747)	0.07 (0.706)	0.400	−0.21 (0.202)	−0.06 (0.743)	−0.02 (0.922)	0.225
Total n-3	**−0.45 (0.029)**	−0.13 (0.515)	−0.23 (0.223)	0.546	−0.32 (0.097)	−0.10 (0.586)	−0.23 (0.220)	0.517	−0.07 (0.951)	−0.05 (0.755)	−0.14 (0.485)	0.506
Linoleic	**−0.43 (0.005)**	−0.08 (0.380)	−0.21 (0.162)	0.349	**−0.38 (0.028)**	0.06 (0.688)	−0.01 (0.954)	0.244	−0.15 (0.445)	0.18 (0.311)	0.19 (0.336)	0.482
Eicosadienoic	0.21 (0.261)	0.00 (0.993)	0.22 (0.260)	0.905	**0.38 (0.038)**	0.24 (0.182)	0.30 (0.144)	0.221	**0.38 (0.047)**	**0.35 (0.046)**	0.22 (0.296)	0.398
Total n-6	−0.31 (0.111)	**−0.43 (0.019)**	−0.14 (0.522)	0.613	−0.29 (0.113)	−0.28 (0.100)	−0.08 (0.676)	0.395	−0.16 (0.391)	−0.01 (0.961)	0.04 (0.870)	0.129
Total PUFA	−0.33 (0.088)	**−0.45 (0.012)**	−0.13 (0.572)	0.873	−0.30 (0.117)	−0.31 (0.071)	0.03 (0.901)	0.908	−0.13 (0.501)	−0.05 (0.794)	0.18 (0.351)	0.828

^1^ β-coefficient (*p*-value) presented for comparisons of lower FA quartiles (Q1–Q3) vs. the highest quartile (Q4). FA, fatty acid; SFA, saturated FA; MUFA, monounsaturated FA; EPA, eicosapentaenoic acid; DHA, docosahexaenoic acid; n-3, omega-3; n-6, omega-6; PUFA, polyunsaturated FA. ^2^ *P*-value for interaction term between HIV status and FA quartile presented. Bolding indicates statistical significance (*p* < 0.05).

**Table 6:** Oleic Acid related differences in growth outcomes among 6–10-year-old perinatally HIV-Infected, HIV-exposed uninfected and HIV-unexposed-uninfected children from Kampala, Uganda.

Outcome	Risk Difference (*p*-Value)			HIV*oleic Acid
	HIV Positive	Exposed	No HIV	*p*-Value ^1^
HAZ				0.1052
Oleic Acid Q1 vs. Q4	**0.73 (0.008)**	0.27 (0.442)	−0.02 (0.950)	
Oleic Acid Q2 vs. Q4	0.24 (0.452)	0.28 (0.551)	**0.61 (0.039)**	
Oleic Acid Q3 vs. Q4	**0.69 (0.003)**	0.44 (0.146)	−0.05 (0.868)	
WAZ				0.0506
Oleic Acid Q1 vs. Q4	**0.95 (0.001)**	0.00 (0.997)	0.07 (0.817)	
Oleic Acid Q2 vs. Q4	0.34 (0.252)	0.30 (0.458)	**0.88 (0.001)**	
Oleic Acid Q3 vs. Q4	**0.73 (0.003)**	0.16 (0.622)	0.47 (0.118)	
BAZ				0.0879
Oleic Acid Q1 vs. Q4	**0.71 (0.009)**	−0.11 (0.730)	−0.01 (0.987)	
Oleic Acid Q2 vs. Q4	0.29 (0.300)	0.42 (0.185)	**0.80 (0.002)**	
Oleic Acid Q3 vs. Q4	0.37 (0.279)	0.04 (0.918)	**0.71 (0.020)**	

^1^*P*-value of interaction term of HIV and oleic acid quartiles in model from Table 5. Bolding indicates statistical significance (*p*< 0.05).

## References

[1] Bazinet R.P., Layé S. (2014). Polyunsaturated Fatty Acids and Their Metabolites in Brain Function and Disease. Nat. Rev. Neurosci..

[2] Eriksson S., Mellstrm D., Strandvik B. (2009). Fatty Acid Pattern in Serum Is Associated with Bone Mineralisation in Healthy 8-Year-Old Children. Br. J. Nutr..

[3] Simopoulos A.P. (2002). The Importance of the Ratio of Omega-6/Omega-3 Essential Fatty Acids. Biomed. Pharmacother..

[4] Imhoff-Kunsch B., Briggs V., Goldenberg T., Ramakrishnan U. (2012). Effect of N-3 Long-Chain Polyunsaturated Fatty Acid Intake During Pregnancy on Maternal, Infant, and Child Health Outcomes: A Systematic Review. Paediatr. Perinat. Epidemiol..

[5] Simopoulos A.P. (1991). Omega-3 Fatty Acids in Health and Disease and in Growth and Development. Am. J. Clin. Nutr..

[6] Jumbe T., Comstock S.S., Hahn S.L., Harris W.S., Kinabo J., Fenton J.I. (2016). Whole Blood Levels of the N-6 Essential Fatty Acid Linoleic Acid Are Inversely Associated with Stunting in 2-to-6 Year Old Tanzanian Children: A Cross-Sectional Study. PLoS ONE.

[7] Adjepong M., Pickens A.C., Jain R., Harris W.S., Annan R.A., Fenton J.I. (2018). Association of Whole Blood N-6 Fatty Acids with Stunting in 2-to-6-Year-Old Northern Ghanaian Children: A Cross-Sectional Study. PLoS ONE.

[8] European Commission (2018). 2018 Nutrition Dashboard for Uganda.

[9] Rukundo P. (2016). Food and Nutrition Situation in a Resource Limited Country—A Literature Review of the Last Decade in Uganda. Int. J. Food Nutr. Sci..

[10] World Health Organization (2014). Comprehensive Implementation Plan on Maternal, Infant and Young Child Nutrition.

[11] Food and Agriculture Organization (2010). Nutrition Country Profiles: Uganda Summary.

[12] Food and Agriculture Organization Uganda at a Glance. http://www.fao.org/uganda/fao-in-uganda/uganda-at-a-glance/en/.

[13] Kabwama S.N., Bahendeka S.K., Wesonga R., Mutungi G., Guwatudde D. (2019). Low Consumption of Fruits and Vegetables among Adults in Uganda: Findings from a Countrywide Cross-Sectional Survey. Arch. Public Health.

[14] Innis S.M. (1991). Essential Fatty Acids in Growth and Development. Prog. Lipid Res..

[15] Heymans H.S. (1981). Essential Fatty Acid Deficiency in Childhood. Tijdschr. Kindergeneeskd..

[16] UNAIDS Aidsinfo. http://aidsinfo.unaids.org.

[17] World Health Organization 30 Years of World Aids Day—2018. https://www.who.int/hiv/mediacentre/news/aids-day2018-timeline/en/.

[18] Arpadi S.M. (2000). Growth Failure in Children with Hiv Infection. J. Acquir. Immune. Defic. Syndr..

[19] Yoon J.M. (2014). Dyslipidemia in Children and Adolescents: When and How to Diagnose and Treat? Pediatr. Gastroenterol. Hepatol. Nutr..

[20] Ram M., Gupte N., Nayak U., Kinikar A.A., Khandave M., Shankar A.V., Sastry J., Bollinger R.C., Gupta A. (2012). Growth Patterns among Hiv-Exposed Infants Receiving Nevirapine Prophylaxis in Pune, India. BMC Infect. Dis..

[21] Bukusuba J., Kikafunda J.K., Whitehead R.G. (2007). Food Security Status in Households of People Living with Hiv/Aids (Plwha) in a Ugandan Urban Setting. Br. J. Nutr..

[22] Giordani B., Novak B., Sikorskii A., Bangirana P., Nakasujja N., Winn B.M., Boivin M.J. (2015). Designing and Evaluating Brain Powered Games for Cognitive Training and Rehabilitation in at-Risk African Children. Glob. Ment. Health (Camb).

[23] Jackson J.B., Musoke P., Fleming T., Guay L.A., Bagenda D., Allen M., Nakabiito C., Sherman J., Bakaki P., Owor M. (2003). Intrapartum and Neonatal Single-Dose Nevirapine Compared with Zidovudine for Prevention of Mother-to-Child Transmission of Hiv-1 in Kampala, Uganda: 18-Month Follow-up of the Hivnet 012 Randomised Trial. Lancet.

[24] Guay L.A., Musoke P., Fleming T., Bagenda D., Allen M., Nakabiito C., Sherman J., Bakaki P., Ducar C., Deseyve M. (1999). Intrapartum and Neonatal Single-Dose Nevirapine Compared with Zidovudine for Prevention of Mother-to-Child Transmission of Hiv-1 in Kampala, Uganda: Hivnet 012 Randomised Trial. Lancet.

[25] World Health Organization (2009). Who Anthroplus for Personal Computers Manual: Software for Assessing Growth of the World’s Children and Adolescents.

[26] Ezeamama A.E., Kizza F.N., Zalwango S.K., Nkwata A.K., Zhang M., Rivera M.L., Sekandi J.N., Kakaire R., Kiwanuka N., Whalen C.C. (2016). Perinatal Hiv Status and Executive Function During School-Age and Adolescence: A Comparative Study of Long-Term Cognitive Capacity among Children from a High Hiv Prevalence Setting. Medicine (Baltimore).

[27] Nkwata A.K., Ezeamama A.E., Kiwanuka N., Kakaire R., Whalen C.C., Kizza F.N., Zalwango S.K., Sekandi J.N. (2016). Psychosocial Adjustment in Perinatally Human Immunodeficiency Virus Infected or Exposed Children—A Retrospective Cohort Study. J. Int. AIDS Soc..

[28] Masood A., Stark K.D., Salem N. (2005). A Simplified and Efficient Method for the Analysis of Fatty Acid Methyl Esters Suitable for Large Clinical Studies. J. Lipid Res..

[29] Bailey R.C., Kamenga M.C., Nsuami M.J., Nieburg P., St Louis M.E. (1999). Growth of Children According to Maternal and Child Hiv, Immunological and Disease Characteristics: A Prospective Cohort Study in Kinshasa, Democratic Republic of Congo. Int. J. Epidemiol..

[30] Marinda E., Humphrey J.H., Iliff P.J., Mutasa K., Nathoo K.J., Piwoz E.G., Moulton L.H., Salama P., Ward B.J., Chidawanyika H. (2007). Child Mortality According to Maternal and Infant Hiv Status in Zimbabwe. Pediatr. Infect. Dis. J..

[31] Filteau S. (2009). The Hiv-Exposed, Uninfected African Child. Trop. Med. Int. Health.

[32] Agostoni C., Zuccotti G.V., Riva E., Decarlis S., Bernardo L., Bruzzese M.G., Giovannini M. (1998). Low Levels of Linoleic Acid in Plasma Total Lipids of Hiv-1 Seropositive Children. J. Am. Coll. Nutr..

[33] Bertin J., Barat C., Méthot S., Tremblay M.J. (2012). Interactions between Prostaglandins, Leukotrienes and Hiv-1: Possible Implications for the Central Nervous System. Retrovirology.

[34] Le Roux S.M., Abrams E.J., Donald K.A., Brittain K., Phillips T.K., Nguyen K.K., Zerbe A., Kroon M., Myer L. (2019). Growth Trajectories of Breastfed South African Hiv-Exposed Uninfected and Hiv-Unexposed Children in the Context of Universal Maternal Antiretroviral Therapy: A Prospective Cohort Study. Lancet Child Adolesc. Health.

[35] Siguel E.N., Chee K.M., Gong J., Schaefer E.J. (1987). Criteria for Essential Fatty Acid Deficiency in Plasma as Assessed by Capillary Column Gas-Liquid Chromatography. Clin. Chem..

[36] Martínez M., Mougan I. (1998). Fatty Acid Composition of Human Brain Phospholipids During Normal Development. J. Neurochem..

[37] Mashavave G., Kuona P., Tinago W., Stray-Pedersen B., Munjoma M., Musarurwa C. (2016). Dried Blood Spot Omega-3 and Omega-6 Long Chain Polyunsaturated Fatty Acid Levels in 7–9 Year Old Zimbabwean Children: A Cross Sectional Study. BMC Clin. Pathol..

[38] Lands B., Bibus D., Stark K.D. (2018). Dynamic Interactions of N-3 and N-6 Fatty Acid Nutrients. Prostaglandins Leukot. Essent. Fatty Acids.

[39] Adjepong M., Colecraft E., Harris W., Marquis G., Yakah W., Fenton J. (2018). Association of Whole Blood Fatty Acids and Growth in Southern Ghanaian Children 2–6 Years of Age. Nutrients.

[40] Holman R.T., Johnson S.B., Hatch T.F. (1982). A Case of Human Linolenic Acid Deficiency Involving Neurological Abnormalities. Am. J. Clin. Nutr..

[41] Smuts C.M., van Stuijvenberg M.E., Witthuhn R.C., Dalton A., Wolmarans P., Swanevelder S.A. (2009). A Randomised Control Trial in Schoolchildren Showed Improvement in Cognitive Function after Consuming a Bread Spread, Containing Fish Flour from a Marine Source. Prostaglandins Leukot. Essent. Fatty Acids.

[42] Swanson B., Keithley J.K. (2014). Fish Oil and Its Effects on Inflammation in Hiv-Infected Persons: A Preliminary Review. OA Altern. Med..

[43] Snyder B., Lederman M.M., Sieg S.F., Palettas M., Kuritzkes D.R., Rodriguez B., Playford M.P., Zidar D., Andrade A., Belury M.A. (2017). Prospective Analysis of Lipid Composition Changes with Antiretroviral Therapy and Immune Activation in Persons Living with Hiv. Pathog. Immun..

[44] Zhou D.T., Nehumba D., Oktedalen O., Marange P., Kodogo V., Gomo Z.A., Esterhuizen T.M., Stray-Pedersen B. (2016). Changes in Lipid Profiles of Hiv (+) Adults over Nine Months at a Harare Hiv Clinic: A Longitudinal Study. Biochem. Res. Int..

[45] Jao J., Kirmse B., Yu C., Qiu Y., Powis K., Nshom E., Epie F., Tih P.M., Sperling R.S., Abrams E.J. (2015). Lower Preprandial Insulin and Altered Fuel Use in Hiv/Antiretroviral-Exposed Infants in Cameroon. J. Clin. Endocrinol. Metab..

[46] Cho J.S., Baek S.H., Kim J.Y., Lee J.H., Kim O.Y. (2014). Serum Phospholipid Monounsaturated Fatty Acid Composition and Δ-9-Desaturase Activity Are Associated with Early Alteration of Fasting Glycemic Status. Nutr. Res..

